# METTL16 and YTHDC1 Regulate Spermatogonial Differentiation via m6A

**DOI:** 10.1111/cpr.13782

**Published:** 2024-11-29

**Authors:** Xueying Gu, Xinyuan Dai, Haifeng Sun, Yilong Lian, Xingxu Huang, Bin Shen, Pengfei Zhang

**Affiliations:** ^1^ State Key Laboratory of Reproductive Medicine and Offspring Health Women's Hospital of Nanjing Medical University, Nanjing Women and Children's Healthcare Hospital, Nanjing Medical University Nanjing China; ^2^ Zhejiang Lab Hangzhou China

**Keywords:** m^6^A, male sterility, *Mettl16*, spermatogonial differentiation, *Ythdc1*

## Abstract

Spermatogenesis is a highly unique and intricate process, finely regulated at multiple levels, including post‐transcriptional regulation. N6‐methyladenosine (m6A), the most prevalent internal modification in eukaryotic mRNA, plays a significant role in transcriptional regulation during spermatogenesis. Previous research indicated extensive m6A modification at each stage of spermatogenesis, but depletion of *Mettl3* and/or *Mettl14* in spermatogenic cells with *Stra8‐Cre* did not reveal any detectable abnormalities up to the stage of elongating spermatids. This suggests the involvement of other methyltransferases in the regulation of m6A modification during spermatogonial differentiation and meiosis. As a METTL3/14‐independent m6A methyltransferase, METTL16 remains insufficiently studied in its roles during spermatogenesis. We report that male mice with *Mettl16* ^*vasa‐cre*^ exhibited significantly smaller testes, accompanied by a progressive loss of spermatogonia after birth. Additionally, the deletion of *Mettl16* in A1 spermatogonia using *Stra8‐Cre* results in a blockade in spermatogonial differentiation. Given YTHDC1's specific recognition for METTL16 target genes, we further investigated the role of YTHDC1 using *Ythdc1‐sKO* mouse model. Our results indicate that *Ythdc1* ^*Stra8‐cre*^ also impairs spermatogonial differentiation, similar to the effects observed in *Mettl16* ^*Stra8‐cre*^ mice. RNA‐seq and m6A‐seq analyses revealed that deletion of either *Mettl6* or *Ythdc1* disrupted the gene expression related to chromosome organisation and segregation, ultimately leading to male infertility. Collectively, this study underscores the essential roles of the m6A writer METTL16 and its reader YTHDC1 in the differentiation of spermatogonia.

## Graphical abstract

The deletion of either *Mettl16* or *Ythdc1* with *Stra8‐Cre* disrupted the gene expression related to chromosome organisation and segregation, resulting in aberrant differentiation of spermatogonia and male infertility.

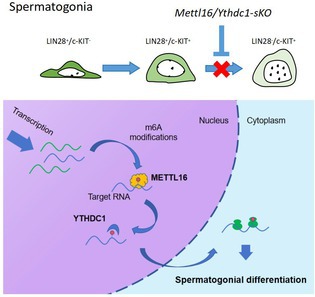

## 1 Introduction

Mammalian spermatogenesis is a highly intricate multi‐step process, which can be broadly divided into three main phases: the mitotic divisions of spermatogonia, the meiotic division of spermatocytes, and the maturation of haploid spermatozoa [1, 2, 3]. In mice, the A single (As) spermatogonia undergo a series of mitotic divisions to generate A paired (Apr) and A aligned (Aal) spermatogonia [4, 5]. These As‐Aal spermatogonia, which harbour stem cell potential, are identified as undifferentiated spermatogonia marked by PLZF and LIN28A [6]. Subsequently, the Aal spermatogonia differentiate into A1 spermatogonia, which begin to express the cell‐surface receptor c‐KIT and initiate an irreversible differentiation process [6, 7]. Following a series of mitotic division, A1 spermatogonia subsequently form type A2, A3, A4, intermediate (In), and B spermatogonia and give rise to meiotic spermatocytes that ultimately produce sperm [1]. To maintain long‐term fertility, these processes are meticulously regulated by complex programs at transcriptional, post‐transcriptional and translational levels [6, 8].

N6‐methyladenosine (m6A), the most prevalent chemical modification in mRNA [9, 10], plays critical roles in various biological processes by regulating the fates of target RNAs including stability, translation and splicing [11, 12, 13]. The m6A modification is dynamic and reversible [12, 13, 14], catalysed by “writer” proteins such as the METTL3 and METTL14 core complex [15, 16], and removed by the “eraser” proteins like ALKBH5 and FTO [17, 18]. This modification exerts its regulatory functions through various “readers,” including YTH domain‐containing proteins (YTHDF1, YTHDF2, YTHDF3, YTHDC1 and YTHDC2), eIF3, HnRNP and IGF2BP [14, 19, 20, 21, 22, 23, 24]. Accumulating evidences show that the regulation of m6A modification is crucial for mammalian spermatogenesis. Germ cell‐specific depletion of METTL3 or METTL14 induces a progressive loss of spermatogonial stem cells (SSCs) [25, 26]. Disruption of the m6A demethylase gene *Alkbh5* results in male infertility in mice due to aberrant metabolism of m6A‐marked mRNAs [18, 27]. Among the m6A reader proteins, YTHDC1 is essential for SSC survival [28], YTHDC2 facilitates proper spermatocyte development [29, 30, 31, 32], and YTHDF2 ensures the timely turnover of phase‐specific transcripts to maintain the proper progression of spermatogenesis [33]. Although most m6A modifications are mediated by the methyltransferase METTL3/METTL14 complex, the combined knockout of both *Mettl3* and *Mettl14* reduced only 55%–65% m6A levels in spermatocytes, without detectable abnormalities up to step 12 of elongating spermatids [26]. Thus, it is reasonable to speculate that multiple methyltransferases are present in germ cells, providing diverse ways to modify transcripts with m6A for essential biological functions.

According to previous RNA‐seq data [34], *Mettl16* and *Ythdc1* were highly expressed in spermatogonia and early MI spermatocytes. METTL16 has been identified as an METTL3/14‐independent RNA methyltransferase responsible for deposition of m6A on specific bulge structures with a consensus sequence UACAGARAA (modified A underlined) in several non‐coding RNAs, including U6 snRNA, *MALAT1*, *XIST* and *MAT2A* [34, 35, 36, 37, 38]. Notably, this unique m6A site can be recognised by the m6A‐binding protein YTHDC1 [35, 37, 39, 40, 41], suggesting the “writer” METTL16 and “reader” YTHDC1 may play the coordinated role in spermatogenesis.

In this study, we demonstrate that the absence of *Mettl16* in early spermatogenic cells resulted in progressive loss of germ cells after birth. The deletion of either *Mettl16* or *Ythdc1* with *Stra8‐Cre* resulted in aberrant differentiation of spermatogonia. Collectively, our studies reveal that the METTL16 and YTHDC1 are essential for the development of spermatogonia and male fertility.

## 2 Materials and Methods

### 2.1 Animal and Cell Lines

The KO first mESC clone for *Mettl16* (C57BL/6N‐*Mettl16* ^tm1a(KOMP)Mbp^/Cmsu) was purchased from CAM‐SU Genomic Resource Center and microinjected into mouse blastocysts. The resulting chimeric mice were crossed with flipper mice to generate *Mettl16‐floxed* mice. The *Ythdc1‐floxed* mice were described before [28]. We generated germ cell‐specific knockout mice by crossing *floxed* mice with *Vasa‐Cre* or *Stra8‐Cre* mice [42, 43], respectively (Figure S1B–E). All mice used in this study were kept at C57BL/6 genetic background and housed under specific pathogen‐free (SPF) conditions. All animal experiments conformed to the guidelines of the Association for Assessment and Accreditation of Laboratory Animal Care in Jiangsu and were approved by the Institutional Animal Care and Use Committee of the Ethics Committee of the Nanjing Medical University (approval No.: IACUC‐14030127‐5).

*Mettl16*‐floxed mouse embryonic stem cells (mESCs) were derived from *Mettl16‐flox* blastocyst and introduced *Cre‐ERT2* to establish a *Mettl16‐iKO* mES cell line by transfected with an appropriate amount of PB‐CAG‐CreERT2‐P2A‐puromycin and PBase by electroporation using Lonza Nucleofector‐4D. To overexpress METTL16‐WT and METTL16‐Mut (the conserved DPPW motif critical for catalysis was mutated to DAAW, PP185/186AA), these above cell lines were electroporated with corresponding overexpression plasmids. To knockout *Mettl16*, the *Mettl16* *‐floxed* mESCs with *Cre‐ERT2* were treated with 4‐Hydroxytamoxifen (4‐OHT) for 72 h.

### 2.2 Haematoxylin and Eosin (H&E) Staining

Testes were fixed in Bouin's buffer or 4% paraformaldehyde (Sigma, 158127) for 24 h. Following dehydration with increasing concentration of ethanol (30%, 50%, 70%, 80%, 90% and 100%), the samples were embedded in paraffin and cut into 5‐μm‐thick sections. Then, the paraffin sections were used for staining with haematoxylin and eosin according to the manufacturer's instructions (C0107). Images were taken using Ni‐E microscope (Nikon).

### 2.3 Immunostaining

For immunofluorescence analysis, sections were boiled in sodium citrate solution buffer (Maxim, MVS‐0066) for 10 min, brought to room temperature, washed in PBS with 0.3% Triton X‐100 (Sangon, A600198‐0500) for 30 min and blocked for 2 h at room temperature. And then, the sections were incubated with the primary antibodies in blocking buffer overnight at 4°C. The following primary antibodies were used in this study: goat anti‐PLZF (1:200, R&D, AF2944), goat anti‐CD117/c‐KIT (1:200, R&D, AF1356), rabbit anti‐DDX4/MVH (1:200, Abcam, AB13840), goat anti‐GFRA1 (1:200, R&D, AF560), rabbit anti‐SCP3 (1:200, Abcam, AB15093), rabbit anti‐LIN28 (1:200, Proteintech, 11724‐1‐AP) and rabbit anti‐YTHDC1 (1:200, Abcam, AB220159). Next day, slides were washed four times for 15 min in PBS with 0.1% Tween‐20 (Sangon, A600560‐0500) and then incubated with secondary antibody pre‐mixed with Hoechst33342 (sigma, B2261) for 2 h at room temperature. The sections were washed in PBS with 0.1% Tween‐20 and then analysed by confocal microscopy (Zeiss).

### 2.4 TUNEL Assay

TUNEL assay was performed with TUNEL BrightRed Apoptosis Detection Kit (Vazyme, A113‐01) according to the manufacturer's instructions. Briefly, paraffin sections were deparaffinised and rehydrated. After incubated with 20 μg/mL Proteinase K for 20 min, washed by PBS 2 times, the sections were incubated with Equilibration Buffer at room temperature for 20 min. Then added 50 μL TdT Buffer to each section and incubated at 37°C for 1 h. The sections were washed 3 times by PBS, incubated with Hoechst33342 for 5 min, mounted with 50% glycerol and imaged with a laser scanning confocal microscope LSM700.

### 2.5 Western Blot Analysis

Total protein lysates were extracted with RIPA lysis buffer. After homogenisation, the suspensions were centrifuged at 12,000 *g* for 30 min at 4°C. The supernatant extracts were used for Western blot. Equal amounts of the protein samples were loaded and separated in a SDS‐PAGE gel and transferred onto the PVDF membranes (Millipore, IPVH00010). After washing by TBST (TBS containing 0.1% Tween‐20), the membranes were blocked with 5% skimmed milk for 2 h and then incubated with primary antibodies overnight at 4°C. On the next day, the membranes were washed four times using TBST and incubated with secondary antibody at room temperature for 2 h. The signals of the target proteins were detected and analysed with a chemiluminescence image analysis system (Tanon5200). Primary antibodies used for Western blot are listed below: anti‐GAPDH (Santa Cruz, sc‐32233), anti‐NASP (Abclone, A6938), anti‐ESCO2 (Proteintech, 23525‐1‐AP) and anti‐METTL16 (CST, #17676).

### 2.6 YTHDC1 RIP and RT‐qPCR

Whole testes were lysed with lysis buffer (150 mM KCl, 20 mM Tris–HCl, pH 7.5, 2 mM EDTA, 0.5% NP‐40, 0.5 mM DTT, 1:100 protease inhibitor cocktail and 1:100 SUPERase•In RNase Inhibitor) for 30 min with gentle rotary shaking at 4°C and then centrifuged at 12,000 *g* for 15 min. The supernatant was pre‐cleared with Dynabeads protein A/G beads for 1 h at 4°C. Meanwhile, 100 μL of protein A/G beads was coated with YTHDC1 antibody (CST, #77422) or Rabbit IgG (CST, #2729) for 2 h at 4°C. 1/10 of pre‐cleared samples were saved as input, and the rest samples were incubated with the pre‐coated beads at 4°C overnight. After washing 6 times with NT2 buffer (200 mM NaCl, 2 mM EDTA, 0.05% NP‐40, 50 mM Tris–HCl pH 7.5, 0.5 mM DTT, 1:1000 protease inhibitor and 1:1000 SUPERase•In RNase Inhibitor), the RNAs bound to the Dynabeads were extracted with TRIzol. The RNA was reverse‐transcribed into cDNA using the HiScript III RT SuperMix for qPCR (Vazyme, R323‐01). For RT‐qPCR, it was performed with AceQ qPCR SYBR Green Master Mix (Vazyme, Q141‐03) in a quantitative PCR instrument (QuantStudio Q7). Primer sequences are listed in Table S1.

### 2.7 RNA‐Seq and Data Analysis

Total RNA was extracted from whole testes by TRIzol (Takara, 9109). mRNA libraries were prepared with VAHTS mRNA‐seq V3 Library Prep Kit for Illumina (Vazyme, NR611) according to the manufacturer's instructions and sequenced on Illumina NovaSeq platform.

Reads were quality‐controlled using FastQC (v0.11.5) and TrimGalore (v0.6.0) with default parameters. rRNAs were removed by using bowtie2 (v2.3.4.1) with “‐‐un‐conc‐gz parameter.” The remaining clean reads were aligned to GRCm38 using HISAT2 (v2.2.1) with “‐‐rna‐strandness RF” parameter. Gene counts were quantified using featureCounts (v1.6.0). Differential expression analysis was performed using R package DESeq2 (v 1.38.3). Genes with adjusted *p* values < 0.05 and fold change > 1.5 were identified as differentially expressed genes (DEGs). Alternative splicing (AS) analysis was performed by using rMATS (v4.0.3beta). Gene ontology (GO) enrichment analysis was performed on DAVID and Metascape databases.

### 2.8 m6A‐Seq and Data Analysis

We performed m6A MeRIP according to the previously described protocol [44]. Briefly, 0.2 fmol spike‐in RNAs (NEB, E1610S) were added to 5 μg total RNA. Then, the mixture was treated with DNase I for 20 min at 37°C and RNA fragmentation buffer for 5 min at 70°C. The fragmented RNA was incubated with m6A antibody (NEB, E1610S) and Protein G Magnetic Beads (Invitrogen, 10003D) for 2 h at 4°C. After incubation, the beads were washed in IP buffer (150 mM NaCl, 10 mM Tris–HCl, pH 7.5, 0.1% NP‐40 in nuclease‐free H_2_O). The RNA was recovered by Zymo RCC‐5 Column and used for library preparation using SMARTer Stranded Total RNA‐Seq Kit v2 (Pico Input Mammalian, Takara, 634413) according to the manufacturer's protocol.

Reads were pre‐processed as above mentioned in mRNA‐Seq. For m6A‐Seq, m6A peaks were called by using R package exomePeak2 (v1.10.0). All BAM files were converted to bigWigs for visualisation in IGV (v2.8.2) by using bamCoverage command in deepTools (v3.5.1). m6A motifs were calculated by findMotifsGenome command in Homer (v4.11). Metagene plot of m6A was performed by using R package Guitar (v2.14.0).

## 3 Results

### 3.1 METTL16 Is Essential for the Maintenance of Spermatogonia

**FIGURE 1:**
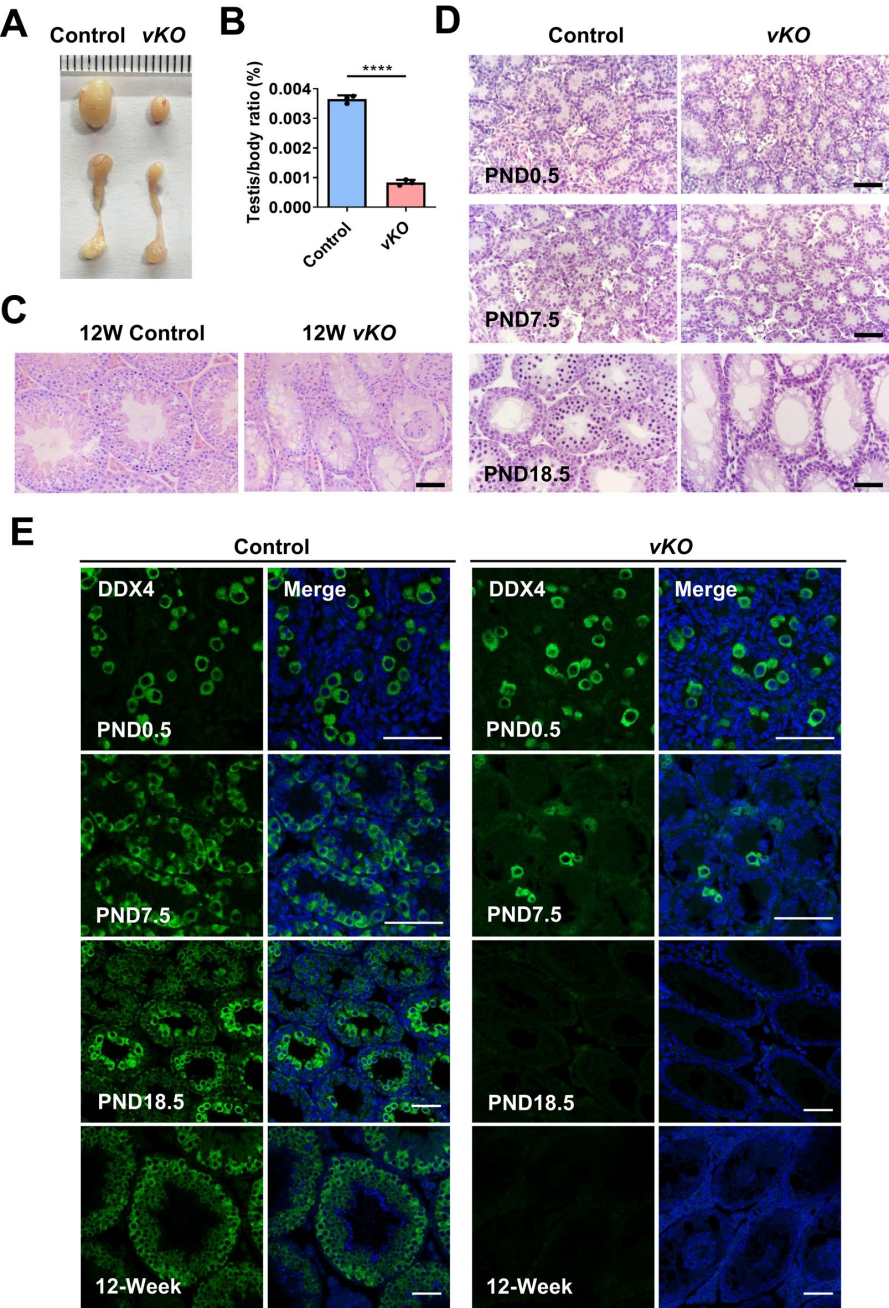
Germ cell‐specific knockout of *Mettl16* causes progressive loss of spermatogonia. (A) Gross morphology of representative testes from adult control and age‐matched *Mettl16* *‐vKO* mice. (B) The testis/body weight ratio of 8W control and *Mettl16* *‐vKO* mice. (C) H&E staining of 8W control and *Mettl16* *‐vKO* testes. Scale bar, 50 μm. (D) H&E staining of control and *Mettl16* *‐vKO* testes at the indicated days. PND, postnatal day. Scale bar, 50 μm. (E) Immunofluorescent staining of DDX4 in control and *Mettl16* *‐vKO* testis at indicated days. Nucleus is stained using Hoechst33342 (blue). Scale bar, 50 μm. Data are presented as means ± SD (*n* = 3 for each group). *p* values were calculated with unpaired one‐tailed Student's *t*‐test (****p < 0.0001).

To investigate the biological functions of METTL6 during spermatogenesis, we first examined its expression pattern in adult mouse testis. Analysis of the published single‐cell RNA‐seq data [43] revealed that *Mettl16* was highly expressed in spermatogonia and early MI spermatocytes (leptotene/zygotene stages), similar to the expression pattern of *Ythdc1* (Figure S1A). Subsequently, we generated a *Mettl16*‐floxed mouse line and crossed it with *Vasa‐Cre* mice to specifically knockout *Mettl16* from embryonic day 15.5 (E15.5) in primordial germ cells, referred to as *Metll16‐vKO* (Figure S1B,C). Morphological analysis demonstrated that adult male mice lacking *Mettl16* were infertile, showing smaller testes and a significantly reduced testis/body ratio (Figure 1A,B). Haematoxylin and Eosin (H&E) staining of testicle sections revealed the absence of germ cells in the testes of adult mice (Figure 1C). We then examined the *Mettl16‐vKO* testes over time to observe the process of germ cell loss. Seminiferous tubules from newborn (PND0.5) *Mettl16‐vKO* males contained prospermatogonia, showing no discernible difference compared to the control group. However, by PND7.5, *Mettl16‐vKO* males exhibited a considerably reduced number of spermatogonia compared to the control group. By PND18.5, *Mettl16‐vKO* males appeared to have lost all germ cells, resulting in a Sertoli cell‐only syndrome (Figure 1D). We further used a germ cell marker (DDX4) to confirm these phenotypes. Consistent with the H&E staining results, *Mettl16‐vKO* males showed obviously fewer spermatogonia than that of control on PND7.5 and lost all germ cells by 2W after birth (Figure 1E and Figure S2). Collectively, these data demonstrate the essential role of METTL16 in the maintenance of spermatogonia. The knockout of *Mettl16* resulted in a progressive loss of germ cells in male mice, ultimately resulting in a Sertoli cell‐only syndrome.

### 3.2 *Mettl16* Is Essential for the Differentiation of Spermatogonia

**FIGURE 2:**
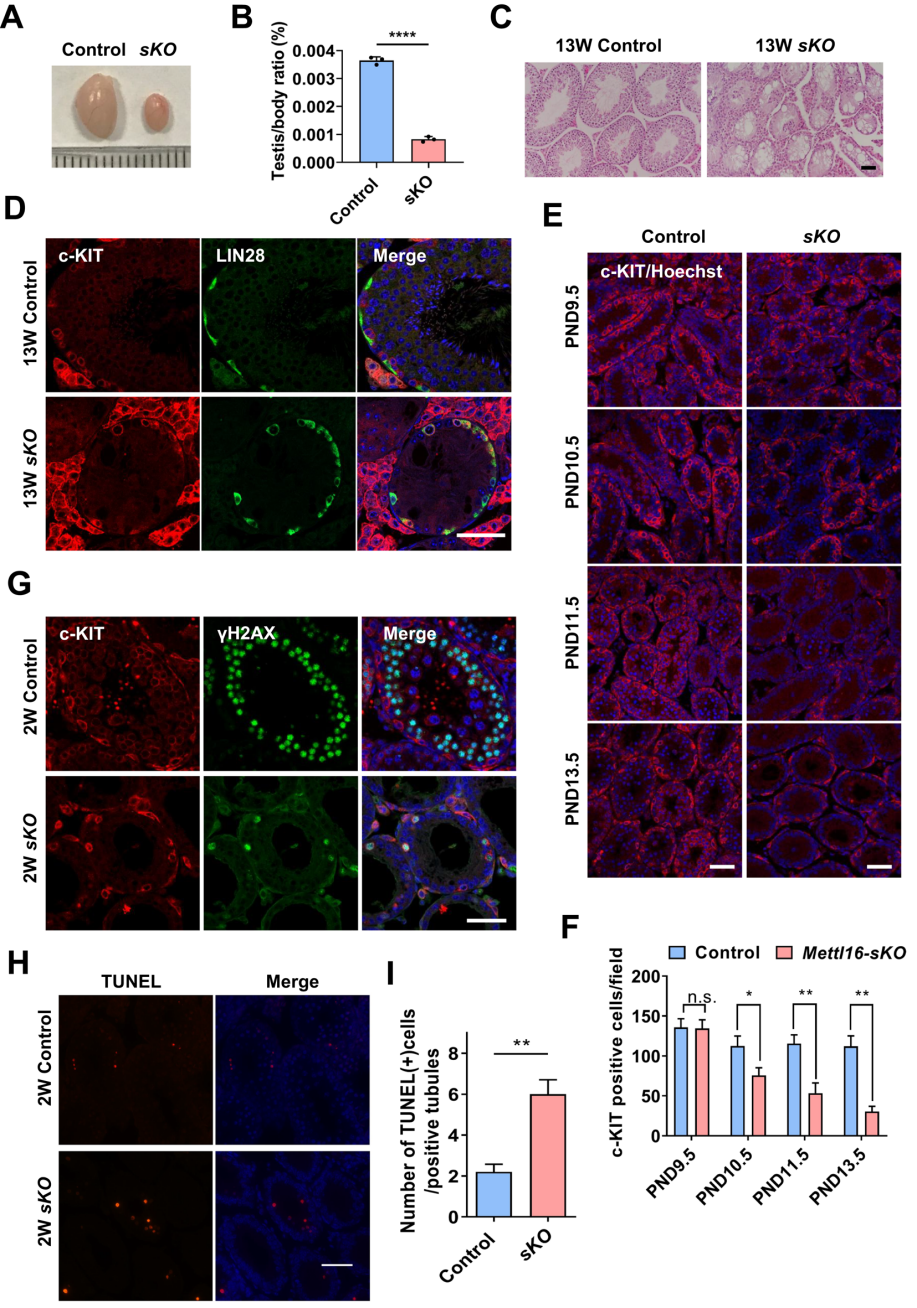
*Mettl16* is essential for the differentiation of spermatogonia. (A) Gross morphology of representative testes from 8W control and age‐matched *Mettl16*‐*sKO* mice. (B) The testis/body weight ratio of 8W control and *Mettl16*‐*sKO* mice. (C) H&E staining of adult control and *Mettl16*‐*sKO* testes. (D) Immunofluorescent staining of c‐KIT and LIN28A in adult control and *Mettl16*‐*sKO* testes. Nucleus is stained using Hoechst33342 (blue). (E) Immunofluorescent staining of c‐KIT in control and *Mettl16*‐*sKO* testes at indicated days. (F) Quantification of c‐KIT‐positive cells in control and *Mettl16*‐*sKO* testes at indicated days. (G) Immunofluorescent staining of γH2AX in 2‐week‐old control and *Mettl16*‐*sKO* testes. (H) TUNEL assay of 2‐week‐old control and *Mettl16*‐*sKO* testes. (I) Quantification of apoptotic cells in 2‐week‐old control and *Mettl16*‐*sKO* testes. Data are presented as means ± SD (*n* = 3 for each group). *p* values were calculated with unpaired one‐tailed Student's *t*‐test (n.s., not significant, *p < 0.05, **p < 0.01, ****p < 0.0001). Scale bar = 50 μm.

Due to the rapid loss of spermatogonia in *Mettl16‐vKO* males, we generated *Mettl16* ^*Stra8‐cre*^ (*Mettl16‐sKO*) to delete *Mettl16* before the initiation of meiotic process to further investigate the role of METTL16 in subsequent spermatogenesis. Through the fertility test, we found the adult *Mettl16‐sKO* males sterile. Compared with control, testes of adult *Mettl16‐sKO* males were smaller (Figure 2A), with significantly reduced testis/body ratio (Figure 2B). *Mettl16‐sKO* males showed vacuolisation along with the absence of germ cell layers (only monolayer seminiferous epithelium) in seminiferous tubules (Figure 2C). Considering the expression of Cre recombinase driven by endogenous *Stra8* promoter initiated from type A1 spermatogonia, we examined the differentiation of spermatogonia in *Mettl16‐sKO* males. As expected, the PLZF‐positive undifferentiated spermatogonia still existed in adult *Mettl16‐sKO* males, while the subsequent germ cells appeared to be absent (Figure S3A), suggesting that deletion of *Mettl16* may impair the differentiation of spermatogonia. We then used undifferentiated (LIN28A) and differentiating (c‐KIT) markers to detect the differentiation state of spermatogonia in *Mettl16‐sKO* male testes. In adult control testes, the undifferentiated spermatogonia with a spindle shape was located on the basal membrane of the seminiferous tubules, and most differentiating spermatogonia appeared oval with only c‐KIT signal (Figure 2D). In *Mettl16‐sKO* testes, almost all spermatogonia were oval‐shaped might be due to the absence of intact seminiferous epithelium, and all c‐KIT‐positive spermatogonia were also labelled by the LIN28A (Figure 2D), indicating that, upon *Mettl16* deletion, the differentiation from LIN28A^+^/c‐KIT^+^ spermatogonia to LIN28A^−^/c‐KIT^+^ spermatogonia, or the survive of c‐KIT^+^ spermatogonia was impaired. To investigate the fate of spermatogonia during differentiation, we examined the changes in c‐KIT‐positive spermatogonia after birth. We observed that the number of these cells significantly decreased starting from PND10.5 in *Mettl16*‐*sKO* testes (Figure 2E,F), suggesting that the differentiating spermatogonia underwent apoptosis. Indeed, in 2‐week‐old *Mettl16*‐*sKO* testes, γH2AX was abnormally expressed in the nucleus of the residual c‐KIT‐positive spermatogonia (Figure 2G), indicating obvious DNA damage in these cells. Consistently, TUNEL analysis showed that the number of TUNEL‐positive cells increased upon METTL16 depletion (Figure 2H,I). Taken together, *Mettl16* is essential for the differentiation of spermatogonia, and knockout of *Mettl16* leads to apoptosis of differentiating spermatogonia.

### 3.3 Depletion of YTHDC1 Impairs Differentiation of Spermatogonia

**FIGURE 3:**
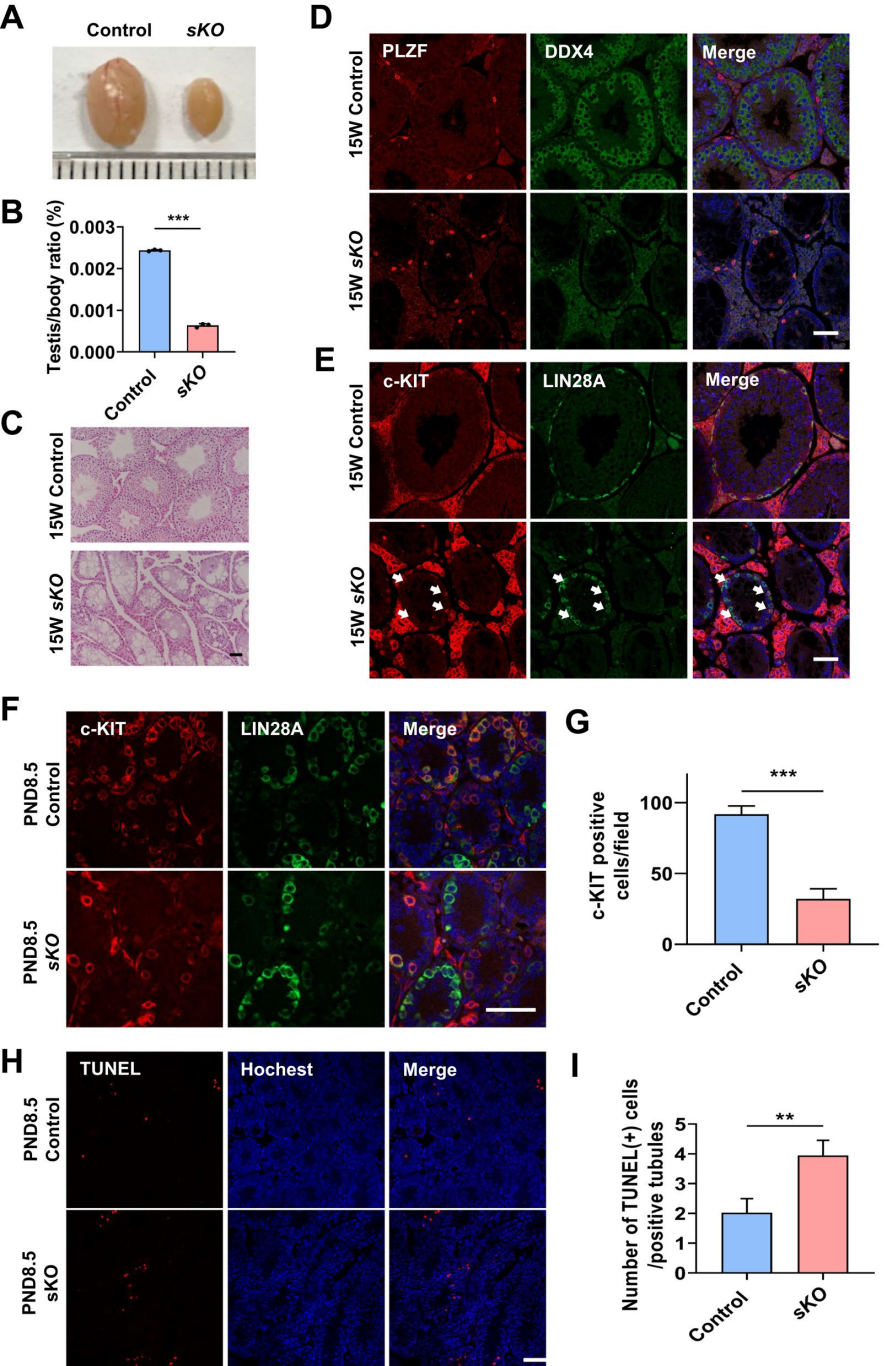
YTHDC1 deletion in germ cells with *Stra8‐Cre* leads to arrested differentiation of spermatogonia. (A) Gross morphology of representative testes from 16W control and age‐matched *Ythdc1*‐*sKO* mice. (B) The testis/body weight ratio of 16W control and *Ythdc1*‐*sKO* mice. (C) H&E staining of adult control and *Ythdc1*‐*sKO* testes. (D) Immunofluorescent staining of PLZF and DDX4 in adult control and *Ythdc1*‐*sKO* testes. (E) Immunofluorescent staining of c‐KIT and LIN28A in adult control and *Ythdc1*‐*sKO* testes. Arrowheads indicate the c‐KIT and LIN28A double‐positive spermatogonia. (F) Immunofluorescent staining of c‐KIT and LIN28A in PND8.5 control and *Ythdc1*‐*sKO* testes. (G) Quantification of c‐KIT‐positive cells in PND8.5 control and *Ythdc1*‐*sKO* testes. (H) TUNEL assay of PND8.5 control and *Ythdc1*‐*sKO* testes. (I) Quantification of apoptotic cells in PND8.5 control and *Ythdc1*‐*sKO* testes. Data are presented as means ± SD (*n* = 3 for each group). *p* values were calculated with unpaired one‐tailed Student's *t*‐test (**p < 0.01, ***p < 0.001). Scale bar = 50 μm.

YTHDC1 has been reported as an important reader of m6A RNA decorated by METTL16 [37]. We noticed that *Ythdc1*‐*vKO* males exhibited a similar phenotype with *Mettl16*‐*vKO* males [28]. Then, we further explored the role of YTHDC1 on differentiation of spermatogonia using the *Ythdc1*‐*sKO* mouse model (Figure S1D,E). As expected, the adult *Ythdc1*‐*sKO* males were sterile, and their testis/body ratio significantly decreased (Figure 3A,B). Testis sections showed vacuolisation along with the absence of germ cell layers in all tubules upon depletion of YTHDC1 (Figure 3C). No noticeable difference was observed in the number of PLZF‐positive undifferentiated spermatogonia between *Ythdc1‐sKO* and control mice (Figure 3D). Similar to *Mettl16‐sKO*, all c‐KIT‐positive germ cells were marked by LIN28 simultaneously in *Ythdc1‐sKO* testes (Figure 3E). However, the number of c‐KIT positive spermatogonia began to significantly decrease from PND8.5 in *Ythdc1‐sKO* testis (Figure 3F,G), earlier than that in *Mettl16‐sKO* testis (Figure 2E,F). TUNEL assay showed that the number of TUNEL‐positive cells significantly increased in PND8.5 *Ythdc1‐sKO* testes (Figure 3H,I), but no significant changes were observed in PND8.5 *Mettl16‐sKO* testes (Figure S3B). Together, *Ythdc1* is essential for the differentiation of spermatogonia, and YTHDC1 depletion induces the earlier impairment during differentiation than ablation of METTL16.

### 3.4 METTL16 or YTHDC1 Depletion Disturbs the Expression of Chromosomal Organisation Genes

**FIGURE 4:**
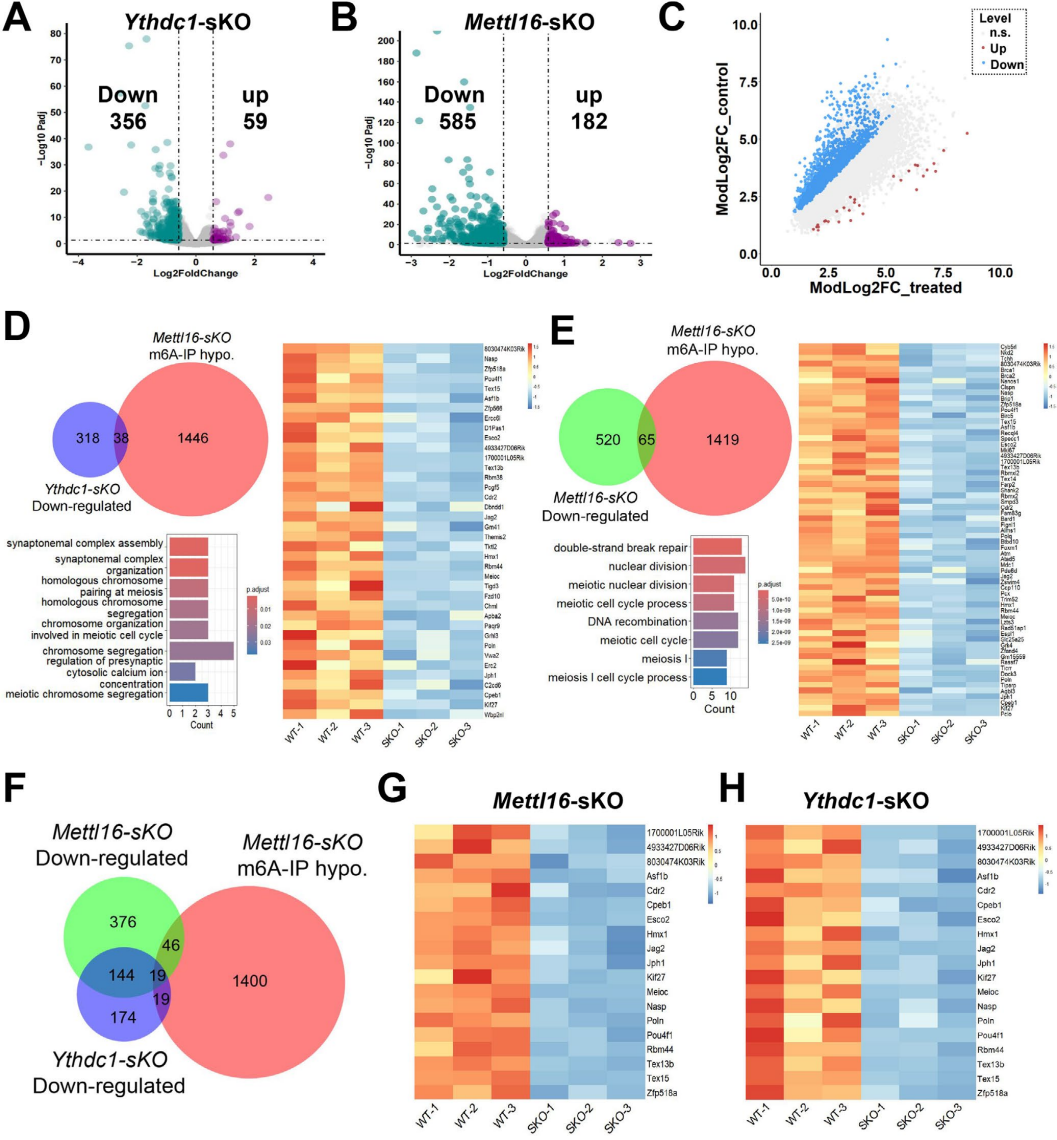
Deletion of METTL3 or YTHDC1 disturbs the transcriptome of testis. (A) Volcano plots showing the differentially expressed genes (DEGs) between PND8.5 control and *Ythdc1*‐*sKO* testes. (B) Volcano plots showing the differentially expressed genes (DEGs)‐ between PND10.5 WT and *Mettl16*‐*sKO* testes. (C) Scatter plots displaying the m6A‐hyper and m6A‐hypo peaks in mRNA of PND10.5 control and *Mettl16*‐*sKO* testes. (D) The overlap between 356 downregulation genes in *Ythdc1*‐*sKO* testes and 1484 m6A‐modified decreased genes in *Mettl16*‐*sKO* testes. (E) The overlap between 585 downregulation genes in *Mettl6*‐*sKO* testes and 1484 m6A‐modified decreased genes in *Mettl16*‐*sKO* testes. (F) Venn diagram showing the 19 overlaps genes among downregulation genes of *Ythdc1*‐*sKO* testes and downregulation genes and m6A‐modified decreased genes of *Mettl6*‐*sKO* testes. (G, H) Expression of the 19 overlaps genes in *Mettl6*‐*sKO* (G) and *Ythdc1*‐*sKO* (H) testes.

To investigate the mechanism of abnormal differentiation of spermatogonia caused by depletion of METTL16 or YTHDC1, we performed m6A‐seq on the testes of *Mettl16*‐*sKO* mice at PND8.5 and RNA‐seq on the testes of *Ythdc1*‐*sKO* mice at PND8.5. At this stage, *Ythdc1*‐*sKO* mice have already displayed abnormal differentiation of spermatogonia, while no obvious differences were observed in *Mettl16*‐*sKO* testes. The RNA‐seq results showed that the deletion of *Ythdc1* induced 356 downregulated genes and 59 upregulated genes (Figure 4A), but only *Mettl16* was downregulated in the PND8.5 *Mettl16*‐*sKO* testes (Figure S4A), which is consistent with the absence of phenotypic differences between *Mettl16‐sKO* and control testes at PND8.5. The IGV results further demonstrated that, similar to *Ythdc1*, the critical exons of *Mettl16* were indeed deleted (Figure S4B). The m6A‐seq revealed that m6A modification level showed no difference between control and *Mettl16*‐*sKO* testes, even on the *Malat1* and *Mat2a* mRNAs, two known targets of METTL16 (Figure S4C). These results suggested that upon the depletion of METTL16, the m6A modification on the target RNAs could still be maintained for a period of time, so no obvious difference was detected in the testes; in contrast, after the depletion of m6A “reader” YTHDC1, the regulation of the target RNA was immediately disrupted, resulting in impaired differentiation of spermatogonia at PND8.5.

We then performed RNA‐seq and m6A‐seq on the testes of *Mettl16*‐*sKO* and control at PND10.5, when the differences in c‐KIT‐positive spermatogonia began to appear in *Mettl16*‐*sKO* testes. Compared with control, *Mettl16*‐*sKO* testes showed 1484 m6A hypomethylation genes and 28 m6A hypermethylation genes, with 585 downregulated genes and 182 up‐regulated genes (Figure 4B,C). The expression of both *Mettl3* and Mettl14 looks normal (Figure S5C,D), indicating that the modification of the 1484 genes with m6A hypomethylation is likely decorated by METTL16. Interestingly, the known targets of METTL16 are not included in these hypomethylation genes, indicating that the installation of m6A modification by METTL16 may be cell‐specific.

To dissect the crosstalk between METTL16 and YTHDC1, we compared the downregulated genes between *Mettl16*‐*sKO* and *Ythdc1*‐*sKO* testes and found 163 common downregulated genes (Figure S5A), accounting for 46.04% of the downregulated genes in *Ythdc1*‐*sKO*, which were related to meiotic cell cycle, cell division and DNA damage response (Figure S5B). Subsequently, we aimed to explore whether the DEGs caused by YTHDC1 or METTL16 depletion are directly regulated via m6A modification. We found that 38 genes showed decreased m6A modification levels out of 356 genes downregulated by *Ythdc1*‐*sKO* (Figure 4D). These 38 genes are mainly involved in chromosomal organisation and segregation. In addition, out of 585 genes downregulated by *Mettl16*‐*sKO*, 65 genes showed a decrease in m6A modification levels and were mainly involved in double‐strand break repair, nuclear division, and meiotic cell cycle process (Figure 4E). This suggests that the absence of METTL16 may lead to abnormal double‐strand break repair in differentiated spermatogonia, disrupting the cell cycle and causing differentiated spermatogonia to enter the apoptotic program. 19 of the 163 common downregulated genes also displayed decreased m6A modification levels (Figure 4F–H), and 7 of these 19 genes are known to be related to DNA metabolism and chromosome assembly (*Nasp*, *Znf518a*, *Asf1b*, *Esco2*, *Poln*, *Tex15*, *Meioc*). The YTHDC1 RIP‐qPCR results revealed that the affinity of the majority of 13 genes to the YTHDC1 protein decreased upon METTL16 depletion, indicating that YTHDC1 is largely able to specifically recognise these METTL16 target genes (Figure S5E).

**FIGURE 5:**
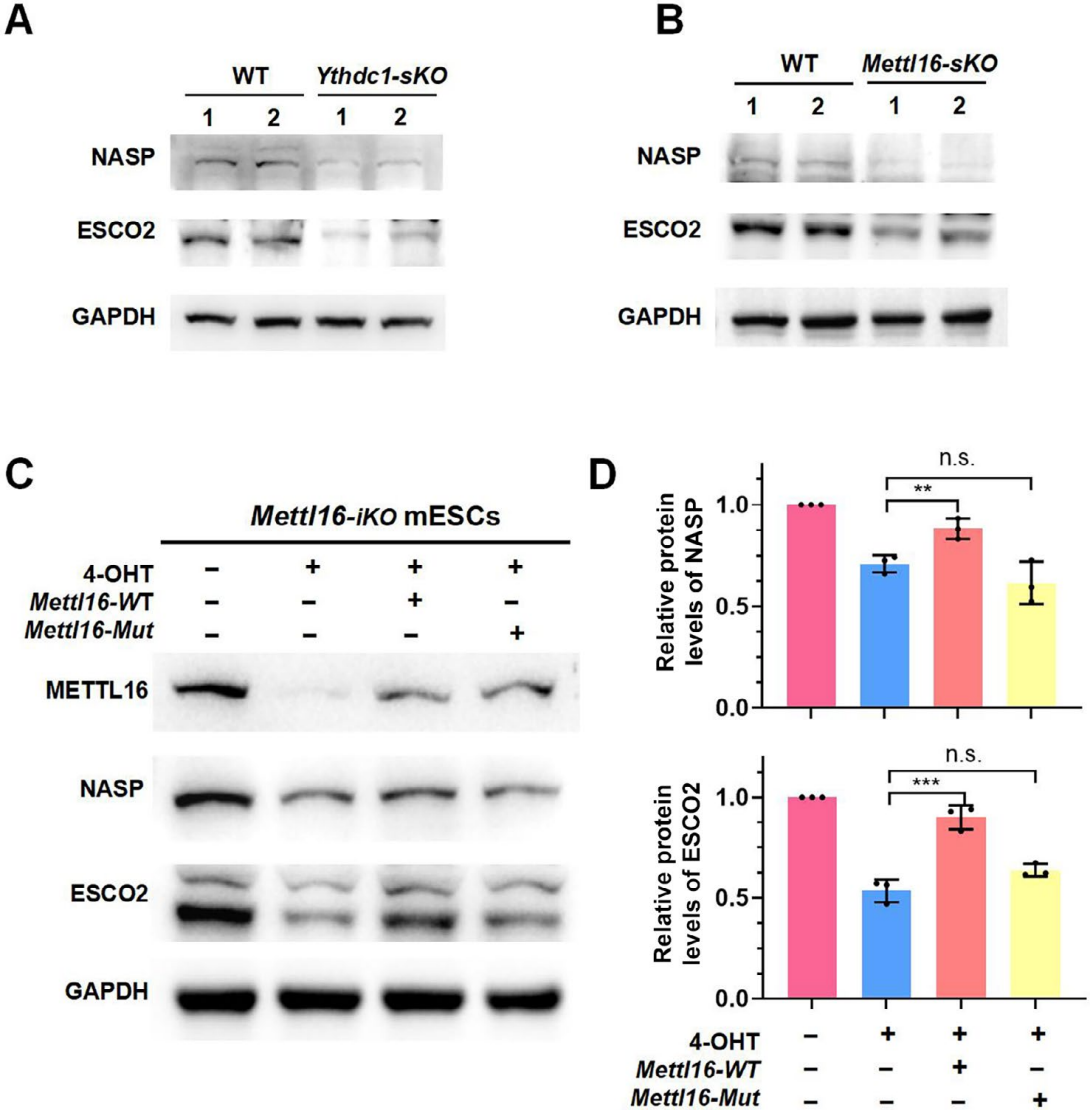
METTL16 and YTHDC1 collaboratively modulate the expression of target genes through an m6A‐dependent mechanism. (A) Western blot of NASP and ESCO2 in testes from PND8.5 WT and *Ythdc1‐sKO* mice. (B) Western blot of NASP and ESCO2 in testes from PND10.5 WT and *Mettl16‐sKO* mice. (C) Validation of the essential role of methyltransferase activity of METTL16 in the regulation of target genes using *Mettl16‐iKO* ESCs. The cells were collected 72 h post 4‐OHT treatment. (D) Quantitative analysis of the protein level of Figure C. Data are presented as means ± SD of *n* = 3 independent biological replicates. Significance was calculated with unpaired two‐tailed Student's *t*‐test (**p < 0.01, ***p < 0.001).

Given that the RNA‐seq results of *Mettl16*‐*sKO* and *Ythdc1*‐*sKO* testes indicate that only a few of DEGs are directly related to the differentiation of spermatogonia, the absence of these two factors is more likely to cause abnormalities in chromosomal organisation and segregation, induce apoptosis in differentiating spermatogonia, and ultimately lead to male infertility. This is consistent with the abnormal expression of γH2AX in the nuclei of the c‐KIT‐positive spermatogonia (Figure 2G). Based on this hypothesis, we chose NASP and ESCO2 for validation, as they are critical for chromosome organisation during spermatogenesis [45, 46, 47, 48]. We conducted additional experiments to assess the protein levels of NASP and ESCO2 in the testes of *Ythdc1‐sKO* and *Mettl16‐sKO* mice. The results showed that the deletion of either *Ythdc1* or *Mettl16* resulted in a decrease in the protein levels of NASP and ESCO2 (Figure 5A,B). These findings indicate that the knockout of *Mettl16* or *Ythdc1* leads to abnormal expression of genes related to chromosome organisation and segregation.

Indeed, it has been reported that METTL16 can exert an m6A‐independent function to facilitate translation and tumorigenesis. To address the concern that the impaired spermatogenesis phenotype observed in *Mettl16‐sKO* mice may not be related to its methylation activity, we conducted additional experiments using embryonic stem cells (ESCs). We generated the *Mettl16‐iKO* mES cell line by deriving ESCs from *Mettl16‐floxed* blastocysts and introducing *Cre‐ERT2*. This approach allowed us to investigate the role of METTL16 enzyme activity by ectopically overexpressing either wild‐type (WT) or catalytically inactive mutant METTL16 (Mut, where the conserved DPPW motif critical for catalysis was mutated to DAAW, PP185/186AA) in these ESCs. We also selected NASP and ESCO2 for validation. The results indicate that overexpression of *Mettl16‐WT* in *Mettl16‐KO* ESCs effectively rescues the downregulation of NASP and ESCO2, whereas *Mettl16‐Mut* does not (Figure 5C,D). These findings suggest that the m6A methylation transferase activity of METTL16 is indeed required for regulating its target genes.

## 4 Discussion

N6‐methyladenosine (m6A) is the most abundant internal decoration of mammalian mRNA and is involved in regulation of nearly every aspect of the RNA life cycle, including RNA splicing, export, stability, and translation [13, 19]. Due to its multifaceted functions, m6A plays essential roles in various cellular, developmental, and pathological processes, including spermatogenesis [26, 33]. Its dysregulation is associated with abnormal spermatogenesis. As an m6A “eraser”, ALKBH5 deficiency could cause male fertility impairment, resulting from apoptosis affecting meiotic metaphase spermatocytes [18]. Loss of either methyltransferase METTL3 or METTL14 in early germ cells leads to dysregulated translation of SSC/progenitor cell proliferation and differentiation factors, causing SSC depletion [26]. Deletion of any one of YTHDC1, YTHDC2 or YTHDF2 also leads to male sterility [28, 31, 32, 33].

Although the m6A methyltransferase complex (MTC), composed of METTL3 and METTL14, has been thought to be the main m6A writer [19], depletion of METTL3 and METTL14 using *Stra8‐Cre* is known to cause 555%–65% decrease in m6A levels in germ cells, without affecting the spermatogonial differentiation and the initiation of meiosis [26]. These observations imply the existence of other m6A methyltransferases in spermatogenesis.

In this study, we found METTL16, an METTL3/14‐independent m6A methyltransferase [40], highly expressed in spermatogonia and early MI spermatocytes. The absence of METTL16 in early germ cells caused progressive loss of spermatogonia within 2 weeks after birth, displaying a Sertoli‐only phenotype. Moreover, deletion of *Mettl16* in type A1 spermatogonia led to the arrest of spermatogonial differentiation, with few of c‐KIT positive spermatogonia observed and almost no spermatocytes present. The co‐immunofluorescence staining results showed that almost all the remaining c‐KIT‐positive spermatogonia still expressed LIN28A, a marker of undifferentiated spermatogonia, and these residual c‐KIT‐positive spermatogonia were abnormally labelled with γH2AX, a marker of DNA double‐strand break. These results indicated that the differentiation of spermatogonia is adversely affected in *Mettl16‐sKO* testes and could not enter the subsequent processes of spermatogenesis. All these evidences suggest that METTL16 is involved in regulation of spermatogonial differentiation.

To identify the reader proteins that work in concert with METTL16, we analysed the phenotypes of multiple reader protein gene knockout mice models that have been reported. We found that the phenotypes resulting from the *Ythdf1/2/3* knockout mice did not match those from the *Mettl16‐sKO* mice [33]. Although the *Ythdc2‐KO* affected meiosis of spermatocytes [29, 31, 32, 49], it appears to be non‐essential for the spermatogonia. Moreover, several studies shown that the impact of YTHDC2 on meiosis requires its intrinsic RNA helicase activity independent of m6A recognition [30, 32, 50]. It has been reported that YTHDC1 can cooperate with METTL16 to maintain SAM homeostasis [37]. We speculate that YTHDC1 may also play an important role in the spermatogonial differentiation. Not surprisingly, the *Ythdc1‐sKO* males showed a similar phenotype with *Mettl16‐sKO* males, including differentiation arrest of spermatogonia and male sterility. Due to the severe phenotype of the adult mice of the two conditional knockout models, we attempted to examine the differentiation of spermatogonia at PND8.5. The phenotypic analysis of PND8.5 *Ythdc1‐sKO* mice showed that the number of differentiating spermatogonia was significantly reduced, whereas the abnormal apoptosis in the seminiferous tubules was increased. However, these injuries were not detected until PND10.5 in *Mettl6‐sKO* mice. We reason that YTHDC1 may recognise wide‐ranging m6A modifications beyond those mediated by METTL16, or that the m6A modification on the target RNAs may still exist for some time to maintain its function after *Mettl16* deletion.

To further reveal the molecular changes caused by the depletion of METTL16 or YTHDC1, we sequenced the whole testicular mRNA of PND8.5 *Mettl6‐sKO* and *Ythdc1‐sKO* testes. The results demonstrated that *Ythdc1*‐*sKO* resulted in the downregulation of 356 transcripts. However, in the PND8.5 *Mettl16*‐*sKO* testes, apart from *Mettl16*, no other transcripts were downregulated. There are two possible explanations for this: first, the impact of *Mettl16* knockout at PND8.5 may not have accumulated sufficiently to manifest biological effects; second, there are much more sertoli cells and undifferentiated spermatogonia compaired with LIN28A and c‐KIT double‐positive type A spermatogonia in the testis, which masks the differential signals emanating from the knockout cells. Nevertheless, no effective method for A1 spermatogonia sorting has been reported, which is also a challenge that we currently encounter and urgently need to solve in the field. Due to the phenotypic differences between PND8.5 and PND10.5 *Mettl16*‐*sKO* testes, we also performed RNA‐seq and m6A‐seq on the PND10.5 *Mettl16*‐*sKO* testes. There are 585 downregulated genes and 1484 genes with decreased m6A modification induced by *Mettl16*‐*sKO*. We found that 19 common downregulated genes in *Mettl16‐sKO* and *Ythdc1*‐*sKO* testes also showed decreased m6A modification levels in *Mettl16‐sKO* testes. Moreover, 13 of these 19 genes are YTHDC1‐targeted and showed decreased binding to reader protein YTHDC1 after *Mettl16‐sKO*. Only a few genes are directly related to the regulation of spermatogonial differentiation among these genes that were downregulated in expression and m6A modification. Instead, these genes are enriched in processes related to chromosome organisation and segregation, which may induce cell cycle arrest of differentiating spermatogonia and ultimately lead to male infertility. Our result is consistent with the findings of a recent study, which demonstrated that methyltransferase activity of METTL16 is needed for spermatogonial differentiation, and METTL16 can regulate the expression of genes involved in DNA replication and cell cycle regulation via m6A [51]. In this study, we explored the reasons behind the abnormal spermatogonial differentiation caused by *Mettl16* or *YTHDC1* deletion from the perspective of m6A methylation modification. We noted that METTL16 has been reported to participate in protein translation regulation by interacting with components of the translation initiation complex [41]. Additionally, we observed that the knockout of *Mettl16* led to changes in the expression of some genes beyond those regulated by m6A, which may be related to the translation regulation function of METTL16. Despite all of this, our results indicate that METTL16 and YTHDC1 play essential roles in the differentiation of spermatogonia.

## Author Contributions

P.Z. and B.S. conceived the project and designed the experiments. X.G. performed mice maintenance and phenotypic analysis with the help of X.D. B.S. performed library preparation for RNA‐seq and m6A‐seq. Y.L. and H.S. performed bioinformatics analyses and data visualisation. X.H. provided input to the project. P.Z. and B.S. wrote the manuscript with inputs from all authors.

## Ethics Statement

The animal study was reviewed and approved by The Institutional Animal Care Committee of Nanjing Medical University.

## Conflicts of Interest

The authors declare no conflicts of interest.

## Supporting information


Table S1.

**FIGURE S1.** (A) RNA expression of *Mettl16* and *Ythdc1* during spermatogenesis, Data from Chen et al., 2019 [43]. Seven subpopulations of germ cells are identitied as follows: stage 1, type A1‐B spermatogonia; stage 2, preleptotene spermatocytes; stage 3, leptotene/zygotene spermatocytes; stage 4, pachytene spermatocytes; stage 5, diplotene spermatocytes to steps 1–2 spermatids; stage 6, steps 3–6 spermatids; stage 7, steps 7–8 spermatids. (B–E) Schematic diagram and genotyping for the*Mettl16* (B, C) and *Ythdc1* (D, E) conditional knock‐out allele.

**FIGURE S2.** Immunofluorescent staining of 2W control and *Mettl16*‐*vKO* testes. Scale bar, 50 μm.

**FIGURE S3.** (A) Immunofluorescent staining adult (13W) control and *Mettl16*‐*sKO* testes. (B) Quantification of TUNEL positive cells in PDN8.5 control and *Mettl16*‐*sKO* testes (n.s., not significant). Scale bar, 50 μm.

**FIGURE S4.** RNA‐seq and m6A‐seq of PND8.5 testes from control and *Mettl16*‐*sKO* mice. (A) Volcano plots showing the differentially expressed genes (DEGs) between PND8.5 control and *Mettl16*‐*sKO* testes. (B) RNA‐seq of the Mettl16 mRNA in PND8.5 testes from control and *Mettl16*‐*sKO* mice. (C) Track views of m6A enrichment on *Malat1, Mat2a, Xist* and *Rbm3* in PND8.5 testes from control and *Mettl16*‐*sKO* mice.

**FIGURE S5.** RNA‐seq of PND8.5 testes from *Ythdc1‐sKO* mice and PND10.5 testes from *Mettl16*‐*sKO* mice. (A) The overlap between 356 downregulation genes in *Ythdc1*‐*sKO* testes and 585 downregulation genes in *Mettl16*‐*sKO* testes. (B) GO analysis of the 163 common downregulated genes between *Ythdc1*‐*sKO* and Mettl16‐*sKO* testes. (C) The RNA expressions of *Mettl3* and *Mettl14* in control and Mettl16‐*sKO* testes. (D) The protein levels of METTL3 and METTL14 in control and Mettl16‐*sKO* testes. (E) YTHDC1‐RIP qPCR in control and PND10.5 *Mettl16‐sKO* testes. Data are presented as means ± SEM of *n* = 2 independent biological replicates.

## Data Availability

The data that support the findings of this study are openly available in Sequence Read Archive (SRA) at https://www.ncbi.nlm.nih.gov/bioproject/PRJNA1000085, reference number PRJNA1000085.
