# Supplementary figures and images for: METTL16 and YTHDC1 Regulate Spermatogonial Differentiation via m6A
Source: Cell Prolif. 2024 Nov 29;58(5):e13782. doi: 10.1111/cpr.13782 (PMC12099213; doi:10.1111/cpr.13782)

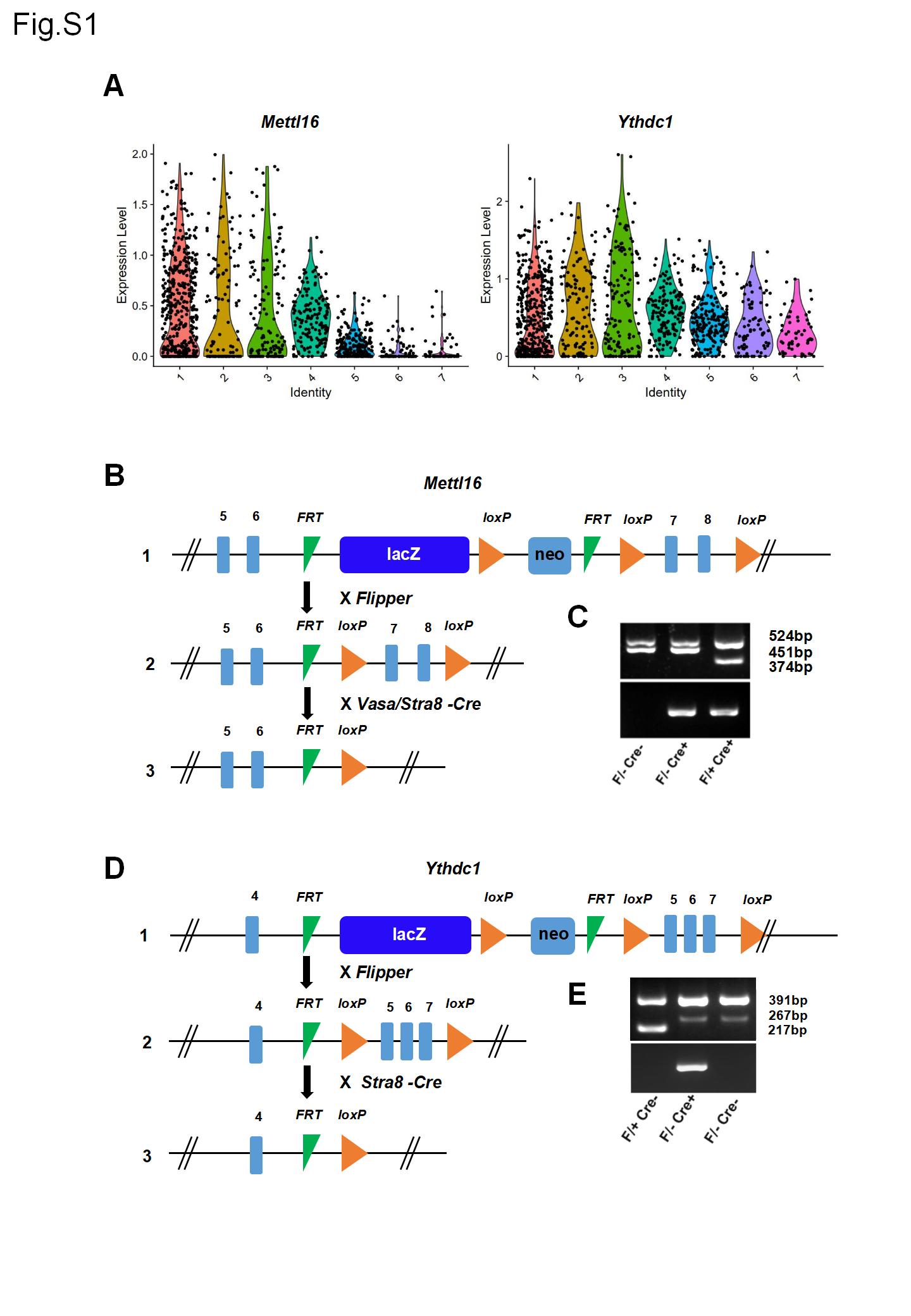

Supplement: Supplementary file 2 — FIGURE S1. (A) RNA expression of Mettl16 and Ythdc1 during spermatogenesis, Data from Chen et al., 2019 [43]. Seven subpopulations of germ cells are identitied as follows: stage 1, type A1‐B spermatogonia; stage 2, preleptotene spermatocytes; stage 3, leptotene/zygotene spermatocytes; stage 4, pachytene spermatocytes; stage 5, diplotene spermatocytes to steps 1–2 spermatids; stage 6, steps 3–6 spermatids; stage 7, steps 7–8 spermatids. (B–E) Schematic diagram and genotyping for theMettl16 (B, C) and Ythdc1 (D, E) conditional knock‐out allele. [file CPR-58-e13782-s004.jpg]

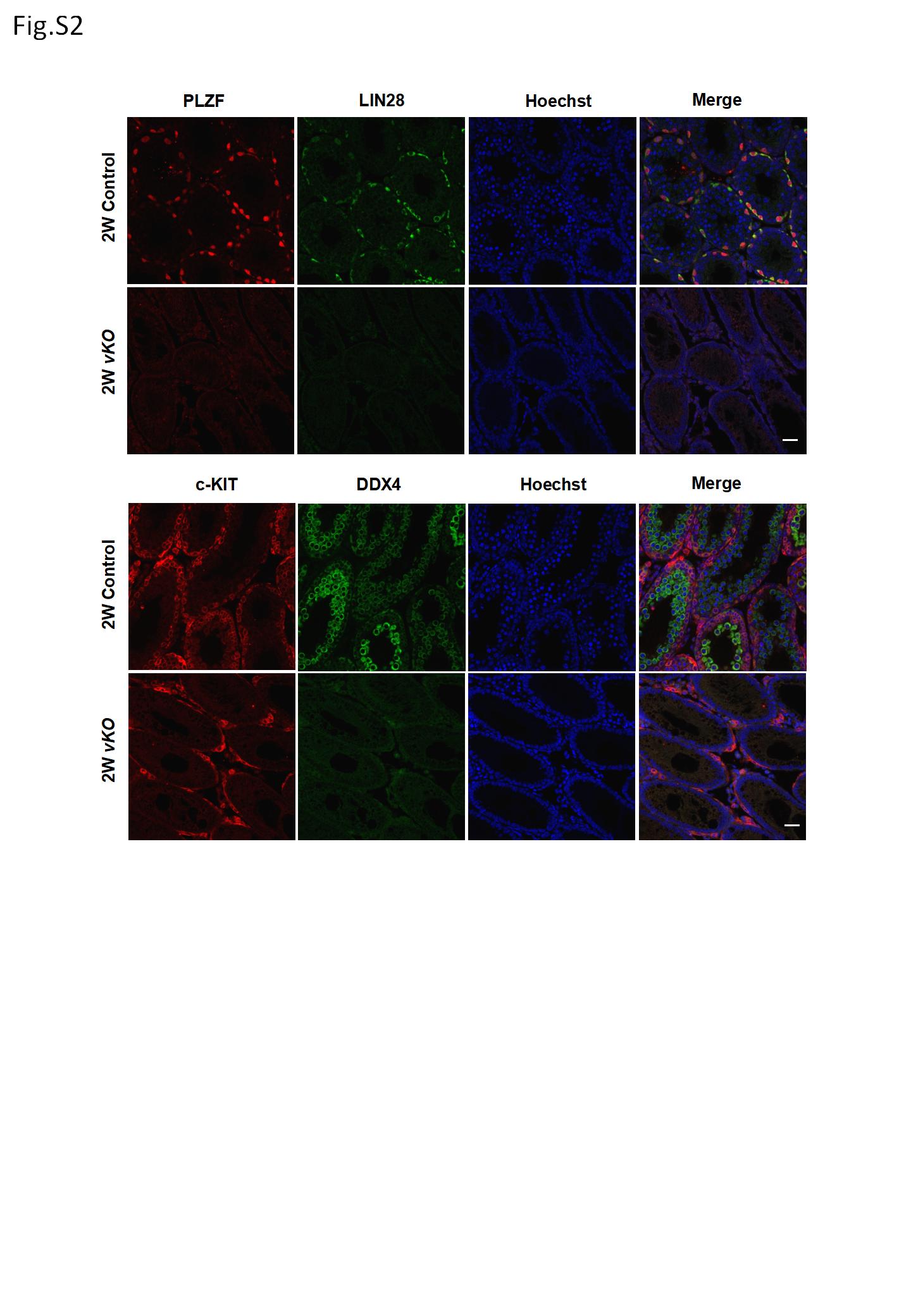

Supplement: Supplementary file 3 — FIGURE S2. Immunofluorescent staining of 2W control and Mettl16‐vKO testes. Scale bar, 50 μm. [file CPR-58-e13782-s001.jpg]

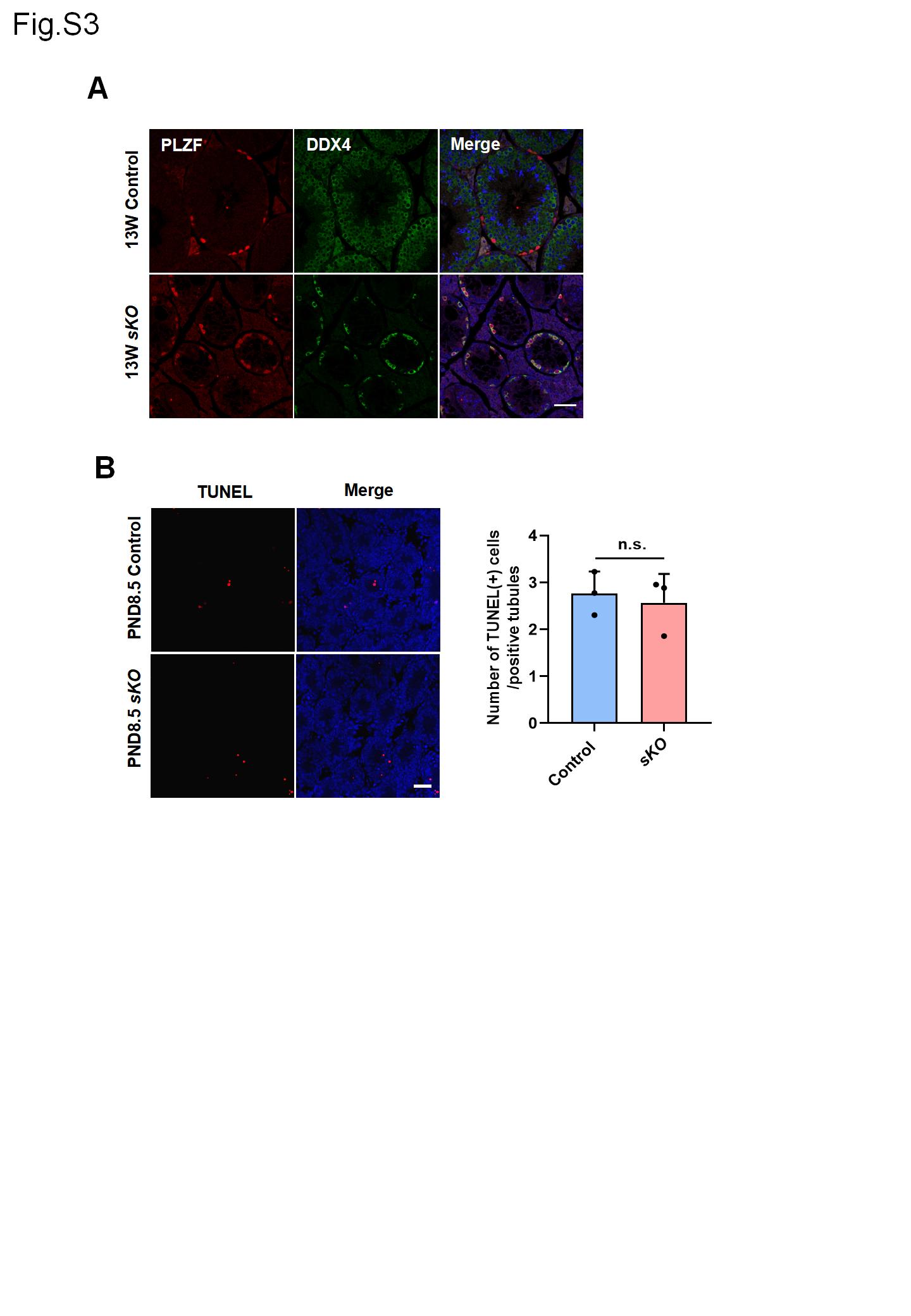

Supplement: Supplementary file 4 — FIGURE S3. (A) Immunofluorescent staining adult (13W) control and Mettl16‐sKO testes. (B) Quantification of TUNEL positive cells in PDN8.5 control and Mettl16‐sKO testes (n.s., not significant). Scale bar, 50 μm. [file CPR-58-e13782-s005.jpg]

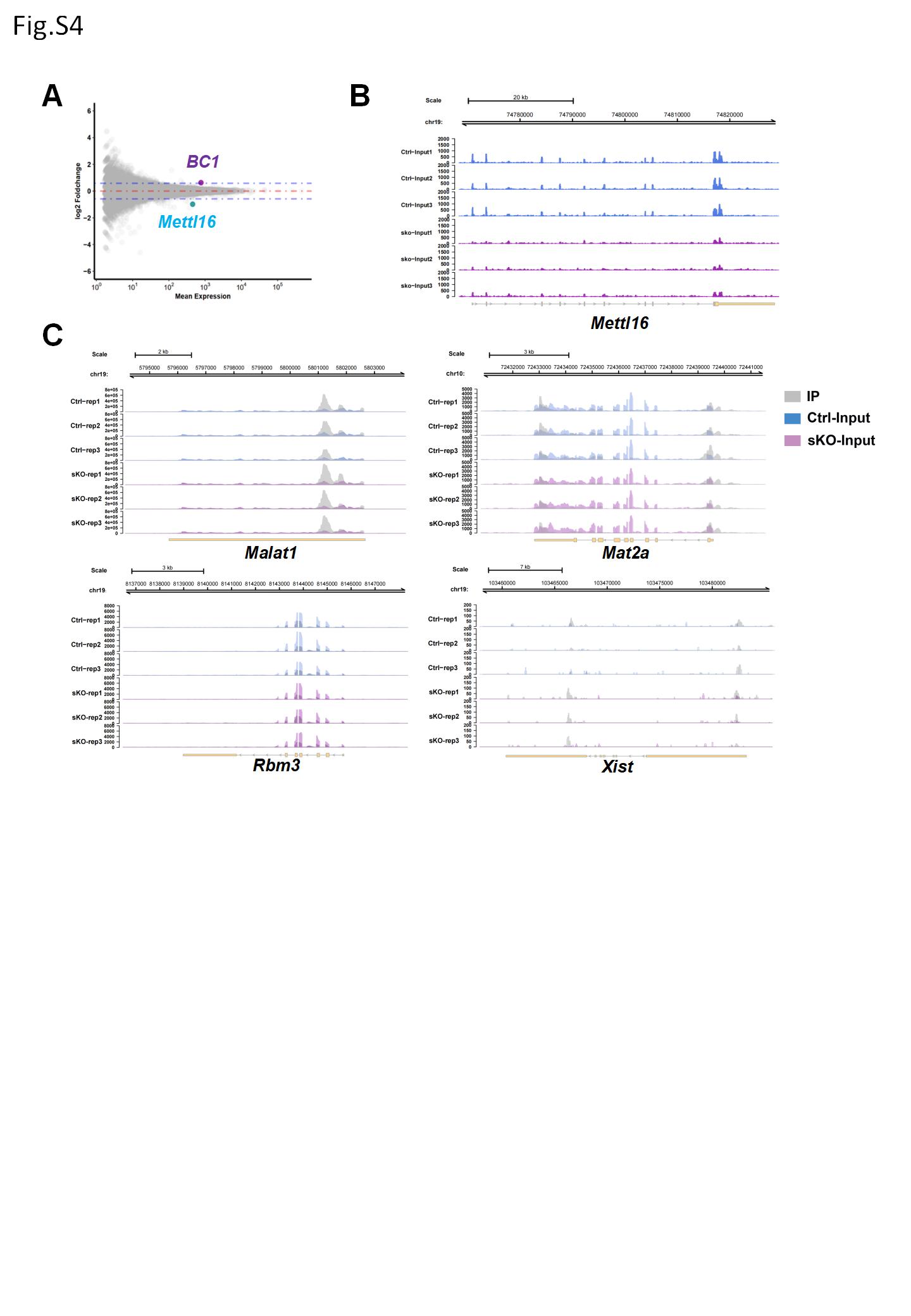

Supplement: Supplementary file 5 — FIGURE S4. RNA‐seq and m6A‐seq of PND8.5 testes from control and Mettl16‐sKO mice. (A) Volcano plots showing the differentially expressed genes (DEGs) between PND8.5 control and Mettl16‐sKO testes. (B) RNA‐seq of the Mettl16 mRNA in PND8.5 testes from control and Mettl16‐sKO mice. (C) Track views of m6A enrichment on Malat1, Mat2a, Xist and Rbm3 in PND8.5 testes from control and Mettl16‐sKO mice. [file CPR-58-e13782-s006.jpg]

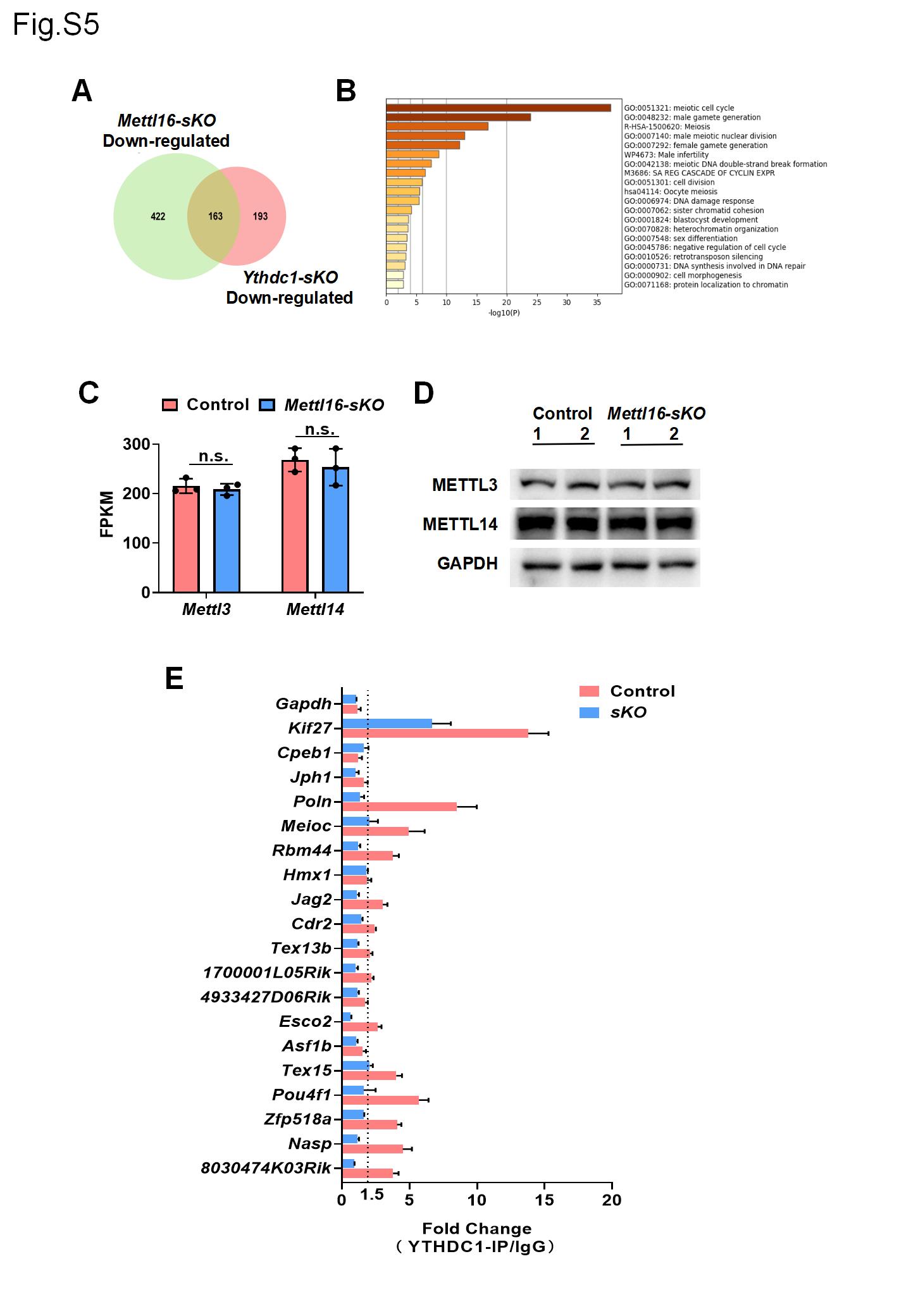

Supplement: Supplementary file 6 — FIGURE S5. RNA‐seq of PND8.5 testes from Ythdc1‐sKO mice and PND10.5 testes from Mettl16‐sKO mice. (A) The overlap between 356 downregulation genes in Ythdc1‐sKO testes and 585 downregulation genes in Mettl16‐sKO testes. (B) GO analysis of the 163 common downregulated genes between Ythdc1‐sKO and Mettl16‐sKO testes. (C) The RNA expressions of Mettl3 and Mettl14 in control and Mettl16‐sKO testes. (D) The protein levels of METTL3 and METTL14 in control and Mettl16‐sKO testes. (E) YTHDC1‐RIP qPCR in control and PND10.5 Mettl16‐sKO testes. Data are presented as means ± SEM of n = 2 independent biological replicates. [file CPR-58-e13782-s002.jpg]
